# The trend of susceptibilities to amphotericin B and fluconazole of *Candida *species from 1999 to 2002 in Taiwan

**DOI:** 10.1186/1471-2334-5-99

**Published:** 2005-11-03

**Authors:** Yun-Liang Yang, Shu-Ying Li, Hsiao-Hsu Cheng, Hsiu-Jung Lo, TSARY Hospitals

**Affiliations:** 1 Department of Biological Science and Technology, National Chiao Tung University, Hsinchu, Taiwan, China; 2 Laboratory for Mycopathogen, Chlamydia and Mycoplasma, Division of Laboratory Research and Development, Center for Disease Control, Taipei, Taiwan, China; 3 Division of Clinical Research, National Health Research Institutes, Miaoli, Taiwan, China

## Abstract

**Background:**

*Candida *species have various degrees of susceptibility to common antifungal drugs. The extent of resistance to amphotericin B and fluconazole of *Candida glabrata *isolates causing candidemia has been reported. Active surveillance may help us to monitor the trend of susceptibility to antifungal drugs and to determine if there is an emerging co-resistance to both drugs of *Candida *species, specifically, of *C. glabrata *in Taiwan.

**Methods:**

The susceptibilities to amphotericin B and fluconazole of *Candida *species collected in 1999 and 2002 of the Taiwan Surveillance of Antimicrobial Resistance of Yeasts (TSARY) were determined by the microdilution method.

**Results:**

The antifungal susceptibilities of 342 and 456 isolates collected from 11 hospitals participating in both TSARY 1999 and TSARY 2002, respectively, have been determined. The resistance rate to amphotericin B has increased from 0.3% in the TSARY1999 to 2.2% in the TSARY 2002. In contrast, the resistance rate to fluconazole has decreased from 8.8% to 2.2%. Nevertheless, significantly more *C. glabrata *isolates were not susceptible to fluconazole in the TSARY 2002 (47.4%) than that in the TSARY 1999 (20.8%). There were 9.8% and 11% of *C. glabrata *isolates having susceptible-dose dependent and resistant phenotype to fluconazole in the TSARY 1999, verse 45.3% and 2.1% in the TSARY 2002.

**Conclusion:**

There was an increase of resistance rate to amphotericin B in *C. glabrata*. On the other hand, although the resistance rate to fluconazole has decreased, almost half of *C. glabrata *isolates were not susceptible to this drug. Hence, continuous monitoring the emerging of co-resistance to both amphotericin B and fluconazole of *Candida *species, specifically, of *C. glabrata*, will be an important early-warning system.

## Background

In the past decade, nosocomial yeast infections have increased globally. In Taiwan, the prevalence of nosocomial candidemia increased 16-fold from 1981 through 1993 [1,2]. In the United States, yeast infections rank as the fourth most common cause of nosocomial bloodstream infection [3,4]. Furthermore, candidemia contribute considerable mortality (31% to 38%), extend the length of hospital stay [5,6], and increase social cost due to lost productivity and disabling complications [7]. Consequently, the Taiwan Surveillance of Antimicrobial Resistance of Yeasts (TSARY) was initiated in 1999 for epidemiological study of yeast infections in Taiwan [8,9]

*Candida *species have various degrees of susceptibility to common antifungal agents. *Candida lusitaniae *is less susceptible to amphotericin B [10] while *Candida krusei *and *Candida glabrata *are less susceptible to fluconazole than other *Candida *species [11-14]. The extent of fluconazole resistance of *C. glabrata *isolates causing candidemia has been reported throughout the United States [15]. Furthermore, *C. glabrata *exhibits variable cross-resistance to the other triazoles, such as voriconazole and posaconazole [13,16-18] and amphotericin B became the next choice. The aim of this study is to investigate the trend of susceptibility to amphotericin B and fluconazole of *Candida *species in Taiwan from 1999 to 2002. Especially, we would like to determine if there is an emerging co-resistance to amphotericin B and fluconazole of *Candida *species, specifically, of *C. glabrata*, in Taiwan.

## Methods

### Organisms and media

Yeast isolates were collected from 11 hospitals participating in both TSARY 1999 and TSARY 2002 [9,19]. Isolates were stored frozen at -70°C in bead containing Microbank cryovials (PRO-LAB Diagnostics, Austin, TX, USA). At the end of the collection period, isolates were kept frozen and transported by an express delivery company to the laboratory at National Health Research Institutes (NHRI) within 24 hours. After their arrival, the isolates were first sub-cultured on to sabouraud dextrose agar (SDA, BBL, Becton Dickinson Cockeysville, MD, USA) to check for purity and identifications. Pure isolates were labeled and stored in vials containing 50% glycerol at -70°C for subsequent analyses.

### Identification

The identification procedure of yeast isolates in the NHRI laboratory was performed as described previously [8]. In general, isolates identified as *C. albicans *by hospitals were first subjected to the germ tube assay in brain heart infusion (BHI, BBL) medium containing 10% fetal bovine serum (JR12003, JRH Biosciences, Australia) at 37°C for 2–3 hours [20]. Isolates positive in germ tube assay were checked for growth at 42°C to differentiate *C. albicans *from *C. dubliniensis *[21]. The VITEK Yeast Biochemical Card (YBC, bioMerieux, St. Louis, MI, USA) was then used to analyze isolates appearing to be negative by the germ tube assay in the NHRI laboratory and isolates identified as non-albicans *Candida *species by the hospitals. API-32C (bioMerieux) was used to assess the NHRI result when the VITEK-YBC showed less than 90% confidence.

### Antifungal susceptibility testing

The minimum inhibitory concentration (MIC) to amphotericin B or fluconazole of each yeast isolate was determined by *in vitro *antifungal susceptibility testing according to the guidelines by the Clinical and Laboratory Standards Institute (CLSI, formerly NCCLS) [22]. The RPMI medium 1640 (31800-022, Invitrogen Corporation, Carlsbad, CA, USA) was used for dilution. Several strains from American Type Culture Collection, namely, ATCC 14053 *C. albicans*, ATCC 9003 *C. glabrata*, ATCC 6258 *C. krusei*, and ATCC20019 *Candida parapsilosis *were used as controls. The growth of each isolate was measured by a Spectra MAX Plus (Molecular Devices Cop. Sunnyvale, California, USA) after 48-hour incubation at 35°C. We also measured the MICs of some randomly-sampled isolates by Etest (AB Biodisk Solna, Sweden) to confirm our results by microdilution.

The interpretation of MICs was conducted according to the guidelines of the CLSI. The MICs to amphotericin B and fluconazole were defined as the lowest concentration of amphotericin B and fluconazole to reduce the turbidity of cells to greater than 95% and 50%, respectively. For amphotericin B, isolates with MIC ≧ 2 μg/ml were considered to be resistant, whereas those with MIC ≦ 1 μg/ml were susceptible. For fluconazole, isolates with MIC ≧ 64 μg/ml were considered resistant, while those with MIC ≦ 8 μg/ml were susceptible. Isolates with MICs between 16 and 32 μg/ml were susceptible-dose dependent. The MICs of 50% and 90% of the total population were defined as MIC_50 _and MIC_90_. For any species with less than ten, the MIC_50 _and MIC_90 _were not showed.

### Database and analysis

The database for this study contained the following characteristic information of each submitted isolate: hospital origin, location and type of the hospital, identification and source of the isolate. The statistic significance of the differences in frequencies and proportions was determined by the chi-square test with Yates' correction. A p value of ≦ 0.05 was considered statistically significant.

## Results

### Distribution of *Candida *species

The distribution of *Candida *species was similar in both surveys. *Candida albicans *was the most common species consisting 37.7% of the total isolates in the TSARY 1999 and 41.2% in the TSARY 2002. *Candida tropicalis *(28.7% in 1999 vs. 28.3% in 2002) and *C. glabrata *(24% in 1999 vs. 20.8% in 2002) were the two most common non-albicans *Candida *species, followed by *C. parapsilosis *(6.4% in 1999 vs. 7.9% in 2002), *C. krusei *(1.2% in 1999 vs. 1.1% in 2002), and others (2% in 1999 vs. 0.7% in 2002). When classified according to the sources, isolates from urine, sputum, blood, wound, and others were 143 (41.8%), 101 (29.5%), 30 (8.8%), 26 (7.6%), and 42 (12.3%), respectively, in the TSARY 1999 verse 186 (40.8%), 111 (24.3%), 50 (11%), 20 (4.4%), and 89 (19.5%), respectively, in the TSARY 2002.

### Susceptibilities to amphotericin B

The susceptibilities to amphotericin B are shown in Table 1. A total of 10 isolates (2.2%) were resistant to amphotericin B in the TSARY 2002, whereas only one (0.3%) in the TSARY 1999 (p < 0.05). Of these 11 amphotericin B resistant isolates, 9 were non-albicans *Candida *species, including 5 *C. krusei*, 3 *C. glabrata*, and 1 *C. tropicalis*. In general, *C. krusei *was less susceptible to amphotericin B than other species.

**Table 1:** The Susceptibilities of *Candida *Species to Amphotericin B

TSARY 1999 MIC μg/ml	cal	ctr	cgl	cpa	ckr	Others	Total
≦ 0.25	19 (14.7)^*a*^	5 (5.1)	5 (6.1)	5 (22.7)	0	3 (42.8)	37 (10.8)
0.5	81 (62.8)	57 (58.2)	52 (63.4)	8 (36.4)	1 (25)	2 (28.6)	201 (58.8)
1	29 (22.5)	36 (36.7)	25 (30.5)	9 (40.9)	2 (50)	2 (28.6)	103 (30.1)
2	0	0	0	0	1 (25)	0	1 (0.3)
Total	129	98	82	22	4	7	342
	
MIC_50 _μg/ml	0.5	0.5	0.5	0.5	1.0	0.5	0.5
MIC_90 _μg/ml	1.0	1.0	1.0	1.0	2.0	1.0	1.0

TSARY 2002 MIC μg/ml	cal	ctr	cgl	cpa	ckr	Others	Total

≦ 0.25	8 (4.2)	1 (0.8)	0	3 (8.3)	0	0	12 (2.6)
0.5	122 (64.9)	70 (54.2)	17 (17.9)	17 (47.2)	1 (20)	1 (33.3)	228 (50)
1	56 (29.8)	57 (44.2)	75 (78.9)	16 (44.5)	0	2 (66.7)	206 (45.2)
2	2 (1.1)	1 (0.8)	3 (3.2)	0	4 (80)	0	10 (2.2)
Total	188	129	95	36	5	3	456
	
MIC_50 _μg/ml	0.5	0.5	1	0.5	2	1	0.5
MIC_90 _μg/ml	1	1	1	1	2	1	1

cal, *C. albicans*; ctr, *C. tropicalis*; cgl, *C. glabrata*; cpa, *C. parapsilosis*; ckr, *C. krusei*

^*a*^number of isolates (%)

### Susceptibilities to fluconazole

The susceptibilities to fluconazole of *Candida *species are shown in Table 2. In the TSARY 1999, a total of 289 (84.5%), 23 (6.7%), and 30 (8.8%) isolates were susceptible, susceptible-dose dependent, and resistant to fluconazole, respectively, whereas in the TSARY 2002, there were 391 (85.5%), 55 (12%), and 10 (2.2%). The MIC_50 _and MIC_90 _of these isolates in the TSARY1999 were 2 μg/ml and 16 μg/ml, respectively, and in the TSARY 2002, they were 1 μg/ml and 16 μg/ml. In the TSARY 1999, 12 (12.2%) *C. tropicalis*, 9 (11%) *C. glabrata*, 5 (3.9%) *C. albicans*, and 4 (100%) *C. krusei*, while in the TSARY 2002, 4 (2.1%) *C. albicans*, 3 (60%) *C. krusei*, and 2 (2.1%) *C. glabrata *were resistant to fluconazole. Fewer isolates in the TSARY 2002 were resistant to fluconazole than that in the TSARY 1999 (p < 0.05). In contrast, more isolates from the TSARY 2002 were susceptible-dose dependent than that in the TSARY 1999 (p < 0.05). Consequently, there were similar portions of isolates susceptible to fluconazole in both surveys. Nevertheless, there were less isolates with MICs ≦ 2 μg/ml to fluconazole in the TSARY 1999 (71.6%, 207/289) than in the TSARY 2002 (81.8%, 320/391) (p < 0.05). Finally, in the TSARY 1999, 82 (24%) of isolates had MICs between 4 and 8 μg/ml to fluconazole. It was down to 71 (15.6%) in the TSARY 2002.

**Table 2:** The Susceptibilities of *Candida *Species to Fluconazole

TSARY 1999 MIC μg/ml	cal	ctr	cgl	cpa	ckr	Others	Total
S	121 (93.8)^*a*^	77 (78.6)	65 (79.2)	21 (95.5)	0	5 (71.4)	289 (84.5)
SDD	3 (2.3)	9 (9.2)	8 (9.8)	1 (4.5)	0	2 (28.6)	23 (6.7)
R	5 (3.9)	12 (12.2)	9 (11)	0	4 (100)	0	30 (8.8)
Total	129	98	82	22	4	7	342
	
MIC_50 _μg/ml	0.25	2	4	1	ND	ND	2
MIC_90 _μg/ml	4	64	64	4	ND	ND	16

TSARY 2002 MIC μg/ml	cal	ctr	cgl	cpa	ckr	Others	Total

S	178 (94.7)	124 (96.1)	50 (52.6)	36 (100)	1 (20)	2 (66.7)	391 (85.8)
SDD	6 (3.2)	5 (3.9)	43 (45.3)	0	1 (20)	0	55 (12)
R	4 (2.1)	0	2 (2.1)	0	3 (60)	1 (33.3)	10 (2.2)
Toal	188	129	95	36	5	3	456
	
MIC_50 _μg/ml	0.25	1	8	1	ND	ND	1
MIC_90 _μg/ml	1	4	32	2	ND	ND	16

cal, *C. albicans*; ctr, *C. tropicalis*; cgl, *C. glabrata*; cpa, *C. parapsilosis*; ckr, *C. krusei*

^*a*^number of isolates (%), S, susceptible; SDD, susceptible-dose dependent; R, resistant ND, not showed due to small number of isolates

## Discussion

The trend of susceptibilities to antifungal drugs of *Candida *species from 1999 to 2002 has been determined in this study. As expected, C. *krusei *had the highest resistance rate to fluconazole among *Candida *species tested, which is consistent with previous reports [9,11]. In contrast, all *C. parapsilosis *isolates were susceptible to fluconazole, which is also consistent with previous reports that *C. parapsilosis *is the most susceptible species to fluconazole [9,18,23,24]. Though the overall resistance rate to fluconazole has decreased from 8.8% to 2.2%, there were significantly more *C. glabrata *isolates not susceptible to fluconazole in the TSARY 2002 than that in the TSARY 1999. Overexpression of *CgCDR1*, *CgCDR2*, and *CgSNQ2*-encoded efflux pumps has been shown to be a major mechanism contributing to the drug resistance [25-27]. It would be interesting to investigate the molecular mechanisms of drug resistance of those clinical resistant isolates.

Recently, triazoles have been developed as the new savior to the issue of drug resistance in Candida infection. Nevertheless, *C. glabrata *exhibits variable cross-resistance among triazoles [9,18,23]. Thus, amphotericin B appears to be the choice for treating systemic infections caused by this species. However, along with the increased use of amphotericin B, 20% and 36% of *C. glabrata *isolates from North America and Latin America, respectively, were reported to be resistant [23]. These data suggest that co-resistance to amphotericin B and fluconazole of *C. glabrata *species may become a problem for clinical therapy worldwide. In our study, we found only three *C. glabrata *isolates resistant to amphotericin B, which is lower than what has been reported. In that study, 20% of *C. glabrata *causing candidemia collected in Taiwan in 2003 were resistant to amphotericin B [16]. Coincidently, more *C. glabrata *isolates in the TSARY 2002 (78.9%) had the MICs of amphotericin B at 1 μg/ml than that in the TSARY 1999 (30.5%). Hence, periodic surveillance is needed to closely monitor the trends of susceptibility to antifungal drugs and for early detection of the newly emerging co-resistance to amphotericin B and fluconazole of *Candida *species, especially, of *C. glabrata*.

## Abbreviations used

TSARY, Taiwan Surveillance of Antimicrobial Resistance of Yeasts; NHRI, National Health Research Institutes; SDA, sabouraud dextrose agar; BHI, brain heart infusion; YBC, Yeast Biochemical Card; MIC, minimum inhibitory concentration; NCCLS, National Committee of Clinical Laboratory Standards; CLSI, Clinical and Laboratory Standards Institute.

## Competing interests

The author(s) declare that they have no competing interests.

## Authors' contributions

YLY and HJL design the study and drafted the manuscript. HHC conduct the experiments with contribution with SYL. TSARY Hospitals provided isolates.

## Pre-publication history

The pre-publication history for this paper can be accessed here:


